# Supplementary figures and images for: The Slx4-Rad1-Rad10 nuclease differentially regulates deletions and duplications induced by a replication fork barrier
Source: PLoS Genet. 2025 May 30;21(5):e1011720. doi: 10.1371/journal.pgen.1011720 (PMC12151478; doi:10.1371/journal.pgen.1011720)

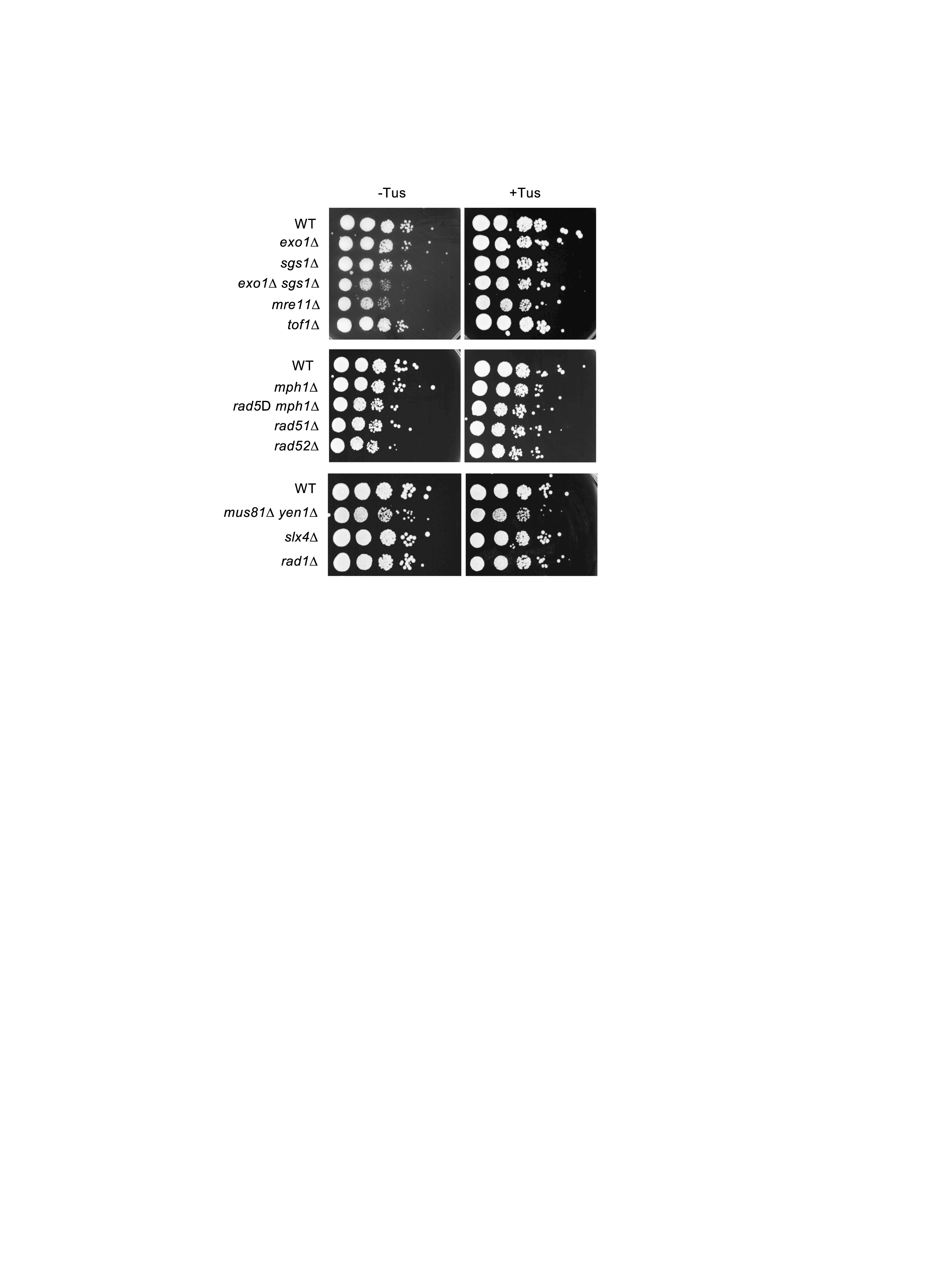

Supplement: S1 Fig — Tenfold serial dilutions of selected strains containing the direct repeat reporter along with the galactose-inducible Tus/Ter system plated on YPAD (-Tus) or YPGAL (+Tus) media and grown for 2 days (YPAD) or 3 days (YPGAL). (TIF) [file pgen.1011720.s001.tif]

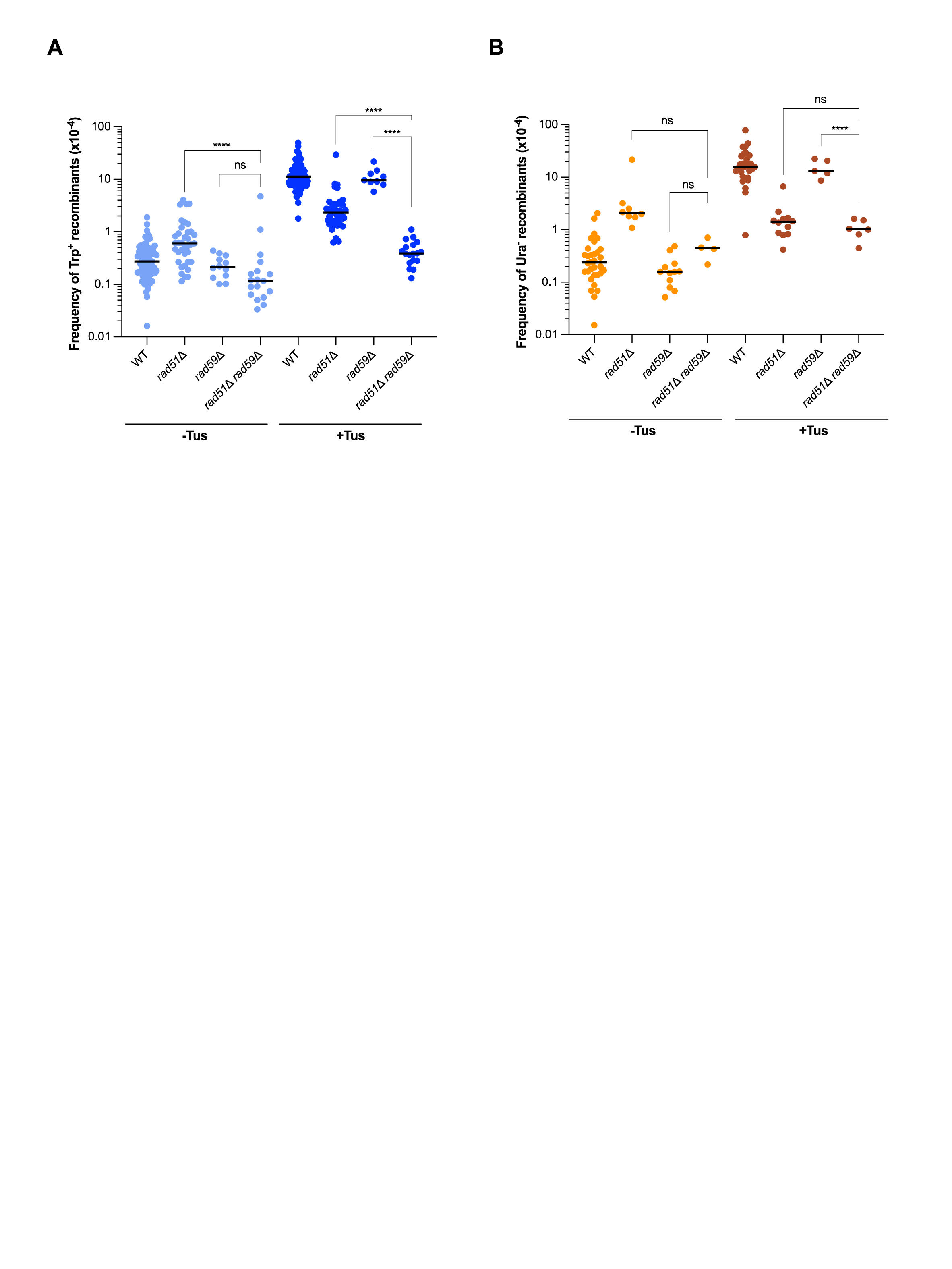

Supplement: S2 Fig — Frequency of Trp+ (A) and Ura- (B) recombinants in WT, rad51Δ, rad59Δ, and rad51Δ rad59Δ strains. Statistical significance was determined by one-way ANOVA on log-transformed data with a Bonferroni post-test. p-values are indicated as follows: ns (not significant) p > 0.05, *p < 0.05, **p < 0.005, ***p < 0.001, ****p < 0.0001. (TIF) [file pgen.1011720.s002.tif]

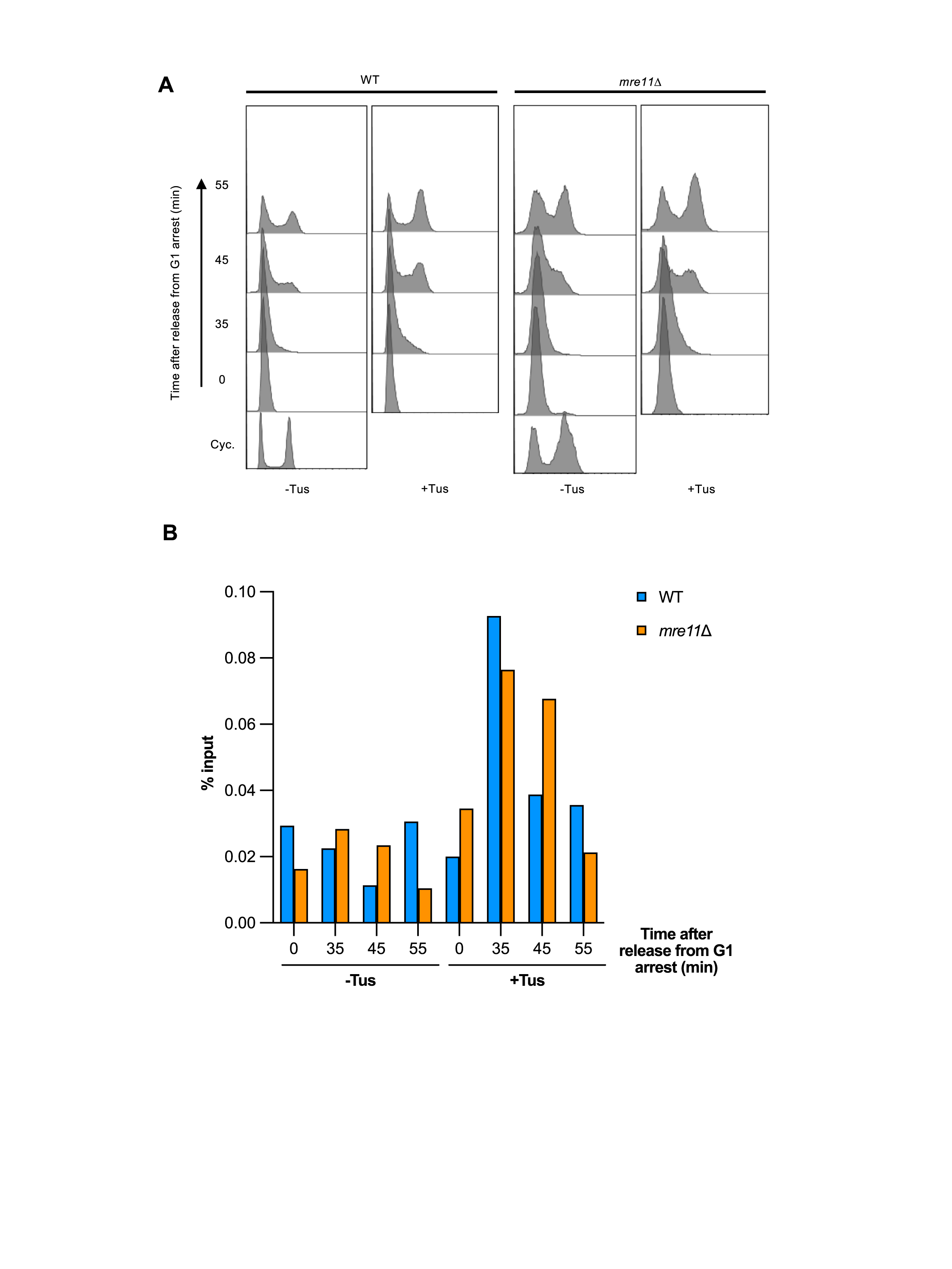

Supplement: S3 Fig — A. FACS profiles for WT and mre11Δ cells after release from G1 arrest -/ + Tus expression. B. ChIP-qPCR for Mcm2–7 using primers 138 bp upstream of the Ter repeats. (TIF) [file pgen.1011720.s003.tif]

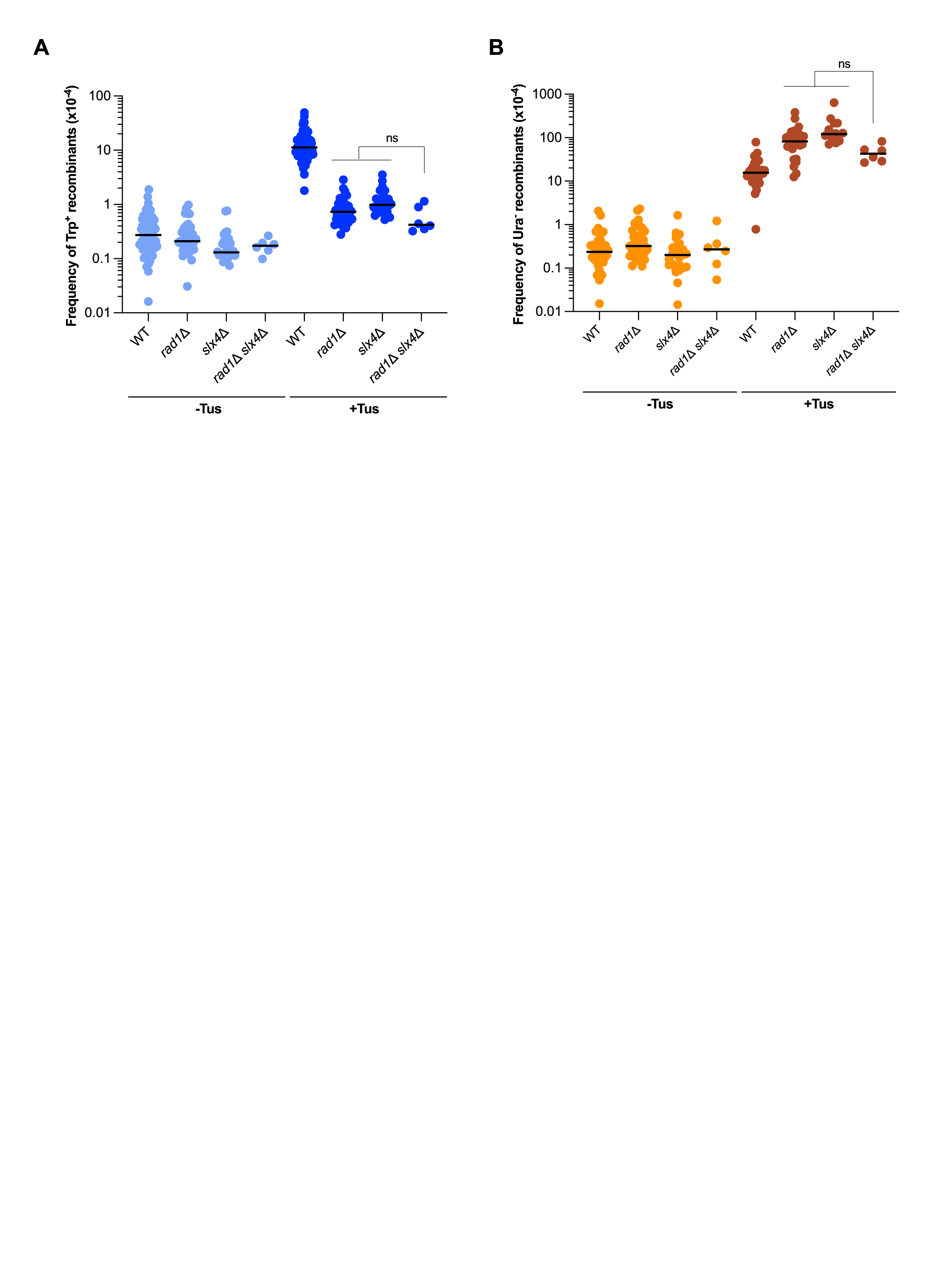

Supplement: S4. Fig — Epistasis between rad1Δ and slx4Δ mutations. Frequency of Trp+ (A) and Ura- (B) recombinants in WT, rad1Δ, slx4Δ, and rad1Δ slx4Δ strains. Statistical significance was determined by one-way ANOVA on log-transformed data with a Bonferroni post-test. p-values are indicated as follows: ns (not significant) p > 0.05, *p < 0.05, **p < 0.005, ***p < 0.001, ****p < 0.0001. (TIF) [file pgen.1011720.s004.tif]

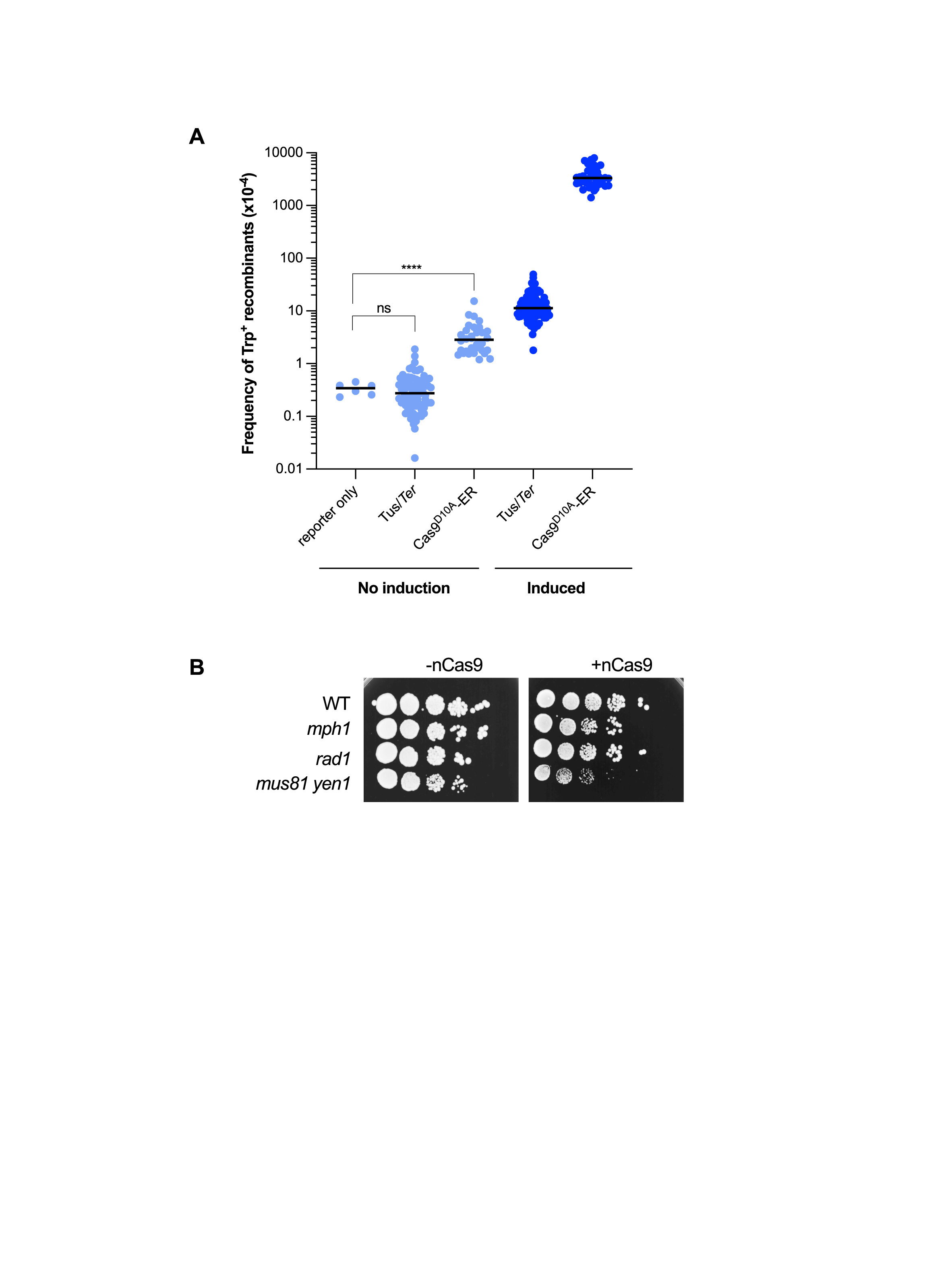

Supplement: S5 Fig — A. Frequencies of Trp+ recombinants in WT strains containing the direct repeat reporter in the absence of replication stress, with the Tus/Ter system, or with the Cas9D10A/gRNA6. B. Ten-fold serial dilutions of the indicated strains with Cas9D10A/gRNA6 plated on YPAD (-nCas9) or YPD + β-estradiol (+ nCas9) media and grown for 2 days (YPAD) or 3 days (YPD + β-estradiol). (TIF) [file pgen.1011720.s005.tif]

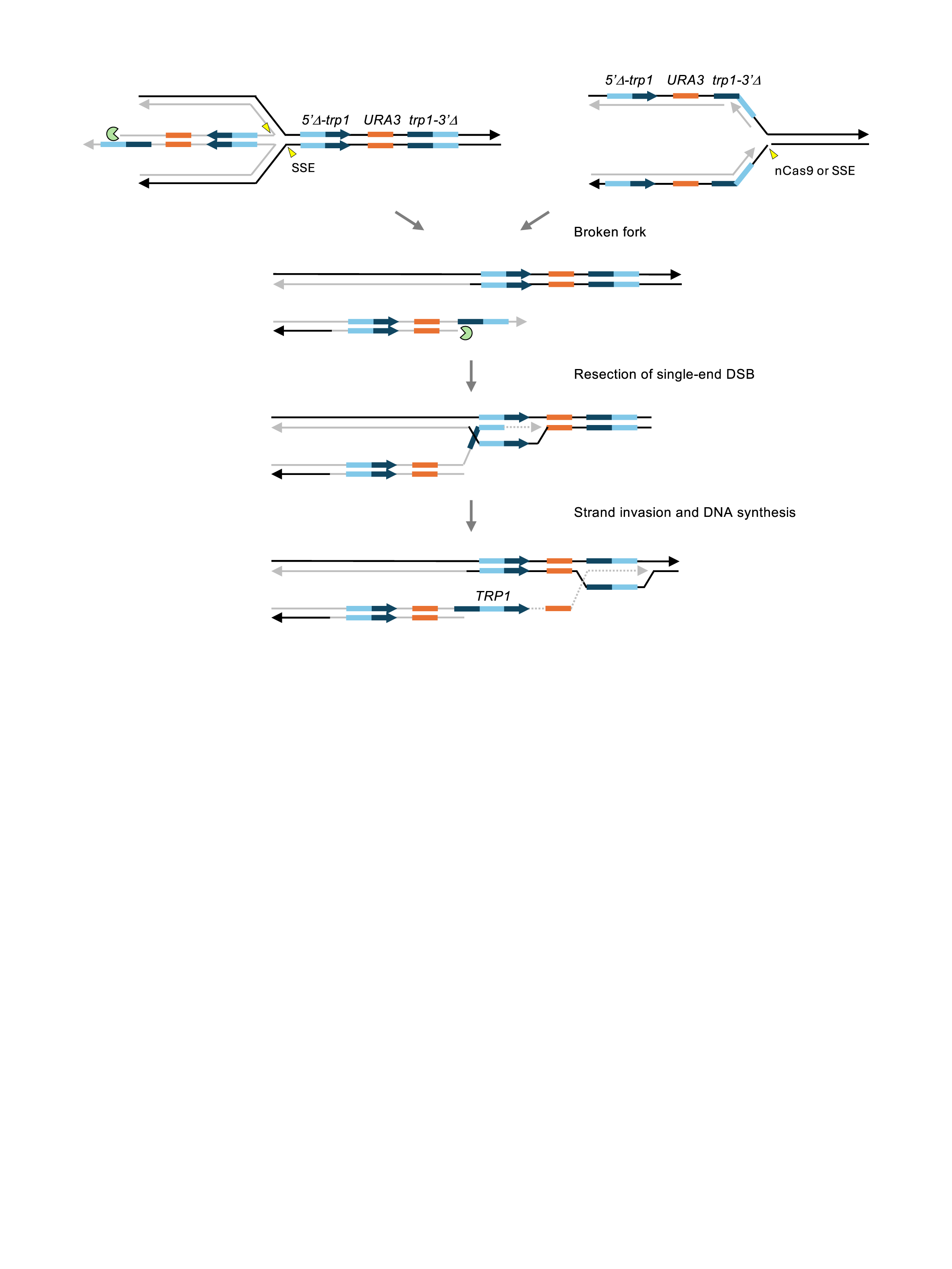

Supplement: S6 Fig — Cleavage of the reversed fork or nicking of the leading strand template downstream of direct repeats would generate a single-ended DSB. Exo1 degradation of the broken arm would create ssDNA for Rad51 assembly and strand invasion. Invasion of the 5′Δ-trp1 repeat by trp1–3′Δ, followed by DNA repair synthesis in the context of a migrating D-loop would reconstitute a functional TRP1 gene (TD). Invasion of the trp1–3′Δ, repeat by 5′Δ-trp1 would have the potential to generate a deletion product. The D-loop could be resolved by an incoming replication fork or BIR to the telomere. The region of homology shared by the repeats is shown in mid-blue, while the 3′ end and 5′ end of TRP1 are shown in dark blue. Parental strand is indicated by black lines, nascent strand by gray lines, and repair-associated DNA synthesis by a dashed gray line. (TIF) [file pgen.1011720.s006.tif]

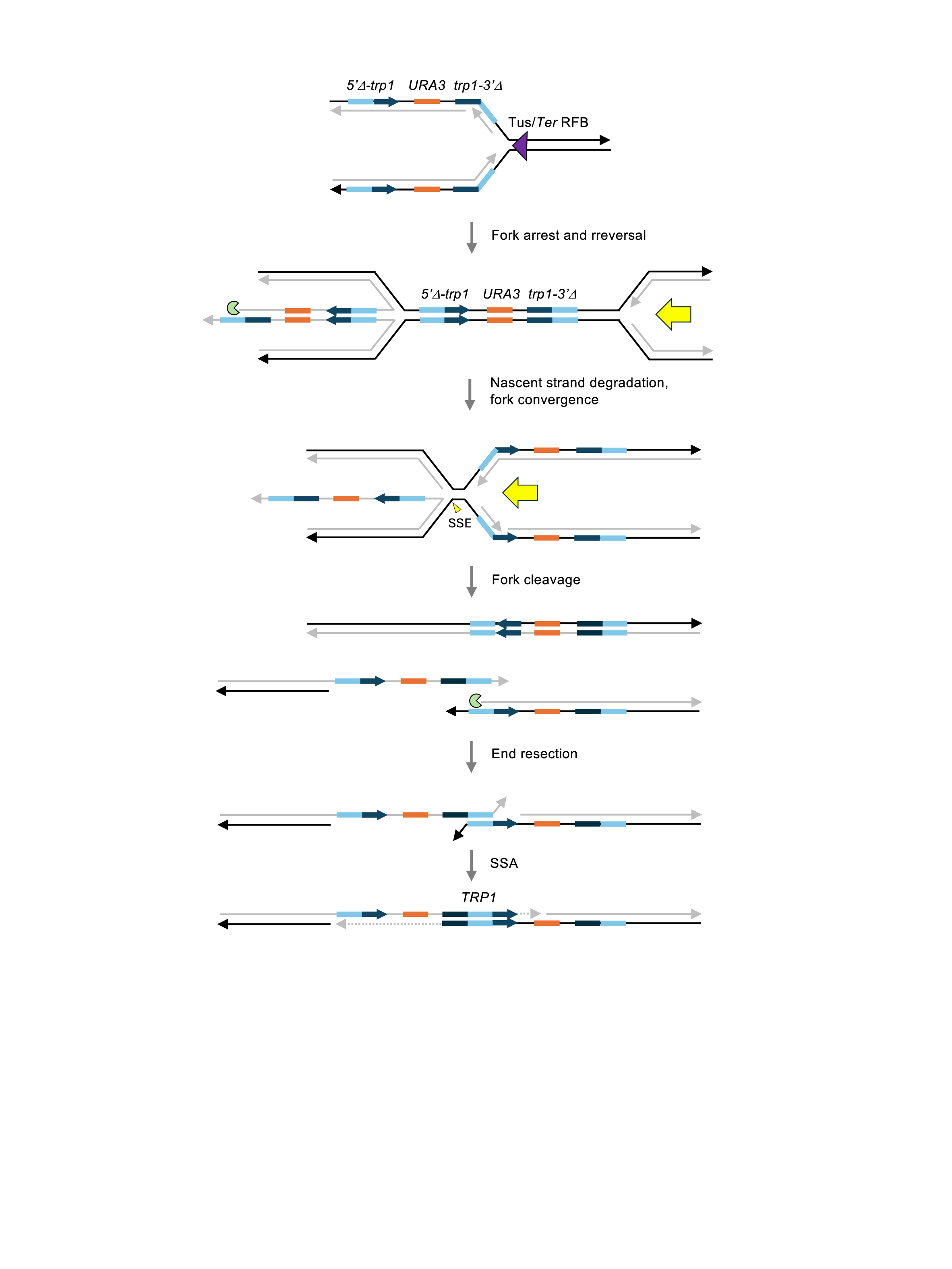

Supplement: S7 Fig — Upon encountering a Tus/Ter barrier, the replication fork could undergo reversal mediated by Mph1. The reversed fork could then be resected through the nuclease activity of Exo1. Alternatively, Exo1 degradation of the nascent lagging strand at the stalled fork would create a ssDNA gap to facilitate pairing of the parental strands, displacing the nascent leading strand. An incoming replication fork could stall at the reversed fork with the over-replicated arm. Cleavage of stalled fork would create a break with overlapping regions of homology to the region of over-replication. Resection of the lagging strand of the broken fork followed by strand annealing would reconstitute a functional copy of TRP1 (TD). The region of homology shared by the repeats is shown in mid-blue, while the 3′ end and 5′ end of TRP1 are shown in dark blue. Parental strand is indicated by black lines, nascent strand by gray lines, and repair-associated DNA synthesis by a dashed gray line. (TIF) [file pgen.1011720.s007.tif]
