# Supplementary material for: The Slx4-Rad1-Rad10 nuclease differentially regulates deletions and duplications induced by a replication fork barrier
Source: PLoS Genet. 2025 May 30;21(5):e1011720. doi: 10.1371/journal.pgen.1011720 (PMC12151478; doi:10.1371/journal.pgen.1011720)
Supplement: S1 Table — (DOCX) [file pgen.1011720.s008.docx]

**S1 Table. Median Trp^+^ recombination frequencies of strains with a Tus/*Ter* block**

|  | **-Tus** | | | **+Tus** | | |  |
| --- | --- | --- | --- | --- | --- | --- | --- |
| **Genotype** | **n** | **Trp^+^ Frequency (x10^-4^)** | **Fold Change (relative to WT)** | **n** | **Trp^+^ Frequency (x10^-4^)** | **Fold Change**  **(relative to WT)** | **Fold Change (+Tus/-Tus)** |
| No Tus/*Ter* | 6 | 0.34 | N/A | 0 | N/A | N/A | N/A |
| WT | 75 | 0.27 | N/A | 79 | 11.3 | N/A | 41.9 |
| *rad52∆* | 27 | 0.02 | -13.5 | 26 | 0.21 | -53.8 | 10.5 |
| *rad51∆* | 37 | 0.6 | 2.2 | 41 | 2.3 | -4.9 | 3.8 |
| *rad59∆* | 12 | 0.21 | -1.3 | 9 | 9.6 | -1.2 | 45.7 |
| *rad51∆ rad59∆* | 18 | 0.12 | -2.3 | 18 | 0.39 | -29.0 | 3.3 |
| *rad5∆* | 17 | 0.22 | -1.2 | 16 | 9.3 | -1.2 | 42.3 |
| *mph1∆* | 28 | 0.35 | 1.3 | 28 | 2.7 | -4.2 | 7.7 |
| *rad5∆ mph1∆* | 23 | 0.40 | 1.5 | 23 | 3.7 | -3.1 | 9.3 |
| *mre11∆* | 42 | 0.28 | 1.0 | 40 | 42.3 | 3.7 | 151.1 |
| *mre11-H125N* | 6 | 0.44 | 1.6 | 6 | 16 | 1.4 | 36.4 |
| *xrs2∆* | 18 | 0.28 | -1.0 | 18 | 50.6 | 4.5 | 180.7 |
| *sae2∆* | 22 | 0.24 | -1.1 | 22 | 11 | -1.0 | 45.8 |
| *dnl4∆* | 5 | 0.32 | 1.2 | 6 | 13.1 | 1.2 | 40.9 |
| *tel1∆* | 12 | 0.49 | 1.8 | 11 | 17.6 | 1.6 | 35.9 |
| *exo1∆* | 18 | 0.48 | 1.8 | 20 | 3.4 | -3.3 | 7.1 |
| *sgs1∆* | 23 | 1.1 | 4.1 | 24 | 184.6 | 16.3 | 167.8 |
| *exo1∆*  *sgs1∆* | 16 | 1.2 | 4.4 | 15 | 16.9 | 1.5 | 14.1 |
| *mus81∆* | 8 | 0.21 | -1.3 | 7 | 7.4 | -1.5 | 35.2 |
| *yen1∆* | 12 | 0.22 | -1.2 | 11 | 8.7 | -1.3 | 39.5 |
| *mus81∆ yen1∆* | 30 | 0.14 | -1.9 | 30 | 2.3 | -4.9 | 16.4 |
| *slx4∆* | 29 | 0.13 | -2.1 | 26 | 0.98 | -11.5 | 7.5 |
| *rad1∆* | 36 | 0.21 | -1.3 | 39 | 0.74 | -15.3 | 3.5 |
| *rad10∆* | 6 | 0.21 | 1.9 | 5 | 0.81 | -14.0 | 3.9 |
| *msh3∆* | 12 | 0.054 | -5.0 | 12 | 1.1 | -10.3 | 20.4 |
| *rad1∆ slx4∆* | 6 | 0.17 | -1.6 | 6 | 0.42 | -26.9 | 2.5 |
| *slx1∆* | 14 | 0.27 | -1.0 | 11 | 11.5 | 1.0 | 42.6 |
| *tof1∆* | 12 | 0.36 | 1.3 | 12 | 32 | 2.8 | 88.9 |
| *csm3∆* | 12 | 0.4 | 1.5 | 12 | 43.7 | 3.9 | 109.3 |
| *scc1-73* | 12 | 0.28 | 1.0 | 12 | 24.1 | 2.1 | 86.1 |
| *rad1∆ rad51∆* | 6 | 0.026 | -10.4 | 6 | 0.047 | -240.4 | 1.8 |
| *rad1∆ rad52∆* | 6 | <0.0007 | NA | 6 | 0.0024 | -4708.3 | NA |
| *mre11∆ rad51∆* | 15 | 0.47 | 1.7 | 15 | 2 | -5.7 | 4.3 |
| WT*+* Exo1OE | 12 | 0.14 | -1.9 | 12 | 7.4 | -1.5 | 52.9 |
| *mre11∆* + Exo1OE | 12 | 0.21 | -1.3 | 12 | 12.5 | 1.1 | 59.5 |
