# Supplementary material for: The Slx4-Rad1-Rad10 nuclease differentially regulates deletions and duplications induced by a replication fork barrier
Source: PLoS Genet. 2025 May 30;21(5):e1011720. doi: 10.1371/journal.pgen.1011720 (PMC12151478; doi:10.1371/journal.pgen.1011720)
Supplement: S2 Table — (DOCX) [file pgen.1011720.s009.docx]

**S2 Table. Median Ura^-^ recombination frequencies of strains with a Tus/*Ter* block**

|  | **-Tus** | | |  | **+Tus** | |  |
| --- | --- | --- | --- | --- | --- | --- | --- |
| **Genotype** | **n** | **Ura^-^ Frequency**  **(x10^-4^)** | **Fold Change (relative to WT)** | **n** | **Ura^-^ Frequency**  **(x10^-4^)** | **Fold Change (relative to WT)** | **Fold Change (+Tus/-Tus)** |
| No  Tus/*Ter* | 6 | 0.22 | N/A | 0 | N/A | N/A | N/A |
| WT | 32 | 0.24 | N/A | 31 | 15.6 | N/A | 65.0 |
| *rad52∆* | 13 | 0.14 | -1.7 | 13 | 0.33 | -47.3 | 2.4 |
| *rad51∆* | 8 | 2.1 | 8.8 | 12 | 1.4 | -11.1 | 0.7 |
| *rad59∆* | 12 | 0.16 | -1.5 | 5 | 13.1 | -1.2 | 81.9 |
| *rad51∆*  *rad59∆* | 4 | 0.44 | 1.8 | 6 | 1 | -15.6 | 2.3 |
| *rad5∆* | 0 | ND | ND | 0 | ND | ND | ND |
| *mph1∆* | 9 | 0.97 | 4.0 | 12 | 1.7 | -9.2 | 1.8 |
| *rad5∆ mph1∆* | 5 | 2.0 | 8.3 | 12 | 3.8 | -4.1 | 1.9 |
| *mre11∆* | 6 | 0.15 | -1.6 | 6 | 65.3 | 4.2 | 435.3 |
| *mre11-H125N* | 6 | 0.47 | 2.0 | 6 | 29.7 | 1.9 | 63.2 |
| *xrs2∆* | 6 | 0.37 | 1.5 | 6 | 112.6 | 7.2 | 304.3 |
| *sae2∆* | 6 | 0.37 | 1.5 | 6 | 23.9 | 1.5 | 64.6 |
| *dnl4∆* | 5 | 0.19 | -1.3 | 6 | 7.7 | -2.0 | 40.5 |
| *tel1∆* | 12 | 0.15 | -1.6 | 11 | 20.5 | 1.3 | 136.7 |
| *exo1∆* | 11 | 0.48 | 2.0 | 11 | 0.36 | -43.3 | 0.8 |
| *sgs1∆* | 16 | 0.7 | 2.9 | 18 | 811.5 | 52 | 1159.3 |
| *exo1∆ sgs1∆* | 5 | 0.2 | -1.2 | 6 | 0.86 | -18.1 | 4.3 |
| *mus81∆* | 0 | ND | ND | 0 | ND | ND | ND |
| *yen1∆* | 0 | ND | ND | 0 | ND | ND | ND |
| *mus81∆ yen1∆* | 12 | 0.1 | -2.4 | 12 | 10.2 | -1.5 | 102.0 |
| *slx4∆* | 25 | 0.2 | -1.2 | 14 | 121.1 | 7.8 | 605.5 |
| *rad1∆* | 36 | 0.32 | 1.3 | 27 | 82.1 | 5.3 | 256.6 |
| *rad10∆* | 6 | 0.52 | 2.2 | 5 | 102.1 | 6.5 | 196.3 |
| *msh3∆* | 9 | 0.2 | -1.2 | 12 | 128.2 | 8.2 | 641 |
| *rad1∆ slx4∆* | 6 | 0.27 | 1.1 | 6 | 43 | 2.8 | 159.3 |
| *slx1∆* | 14 | 0.25 | 1.0 | 12 | 20.5 | 1.3 | 82.0 |
| *tof1∆* | 10 | 2.8 | 11.7 | 12 | 27 | 1.7 | 9.6 |
| *csm3∆* | 9 | 1.7 | 7.1 | 12 | 31.6 | 2.0 | 18.6 |
| *scc1-73* | 12 | 0.38 | 1.6 | 11 | 23.5 | 1.5 | 61.8 |
| *rad1∆*  *rad51∆* | 6 | 0.52 | 2.2 | 6 | 0.27 | -57.8 | 0.5 |
| *rad1∆*  *rad52∆* | 6 | 0.048 | -5.0 | 6 | 0.15 | -104.0 | 3.1 |
| *mre11∆*  *rad51∆* | 15 | 0.48 | 2.0 | 15 | 0.79 | -19.7 | 1.6 |
| WT*+* Exo1OE | 12 | 0.42 | 1.8 | 12 | 14.4 | -1.1 | 34.3 |
| *mre11∆* + Exo1OE | 12 | 0.23 | -1.0 | 12 | 24.8 | 1.6 | 107.8 |
