# Supplementary material for: The Slx4-Rad1-Rad10 nuclease differentially regulates deletions and duplications induced by a replication fork barrier
Source: PLoS Genet. 2025 May 30;21(5):e1011720. doi: 10.1371/journal.pgen.1011720 (PMC12151478; doi:10.1371/journal.pgen.1011720)
Supplement: S3 Table — (DOCX) [file pgen.1011720.s010.docx]

**S3A Table. Median Cas9^D10A^-induced Trp^+^ recombination frequencies**

|  | **Non-induced** | | | **Induced** | | |
| --- | --- | --- | --- | --- | --- | --- |
| **Genotype** | **n** | **Trp^+^ Frequency (x10^-4^)** | **Fold Change (relative to WT)** | **n** | **Trp^+^ Frequency (x10^-4^)** | **Fold Change**  **(relative to WT)** |
| WT | 32 | 2.8 | N/A | 36 | 3328.6 | N/A |
| *mph1∆* | 6 | 5.1 | 1.8 | 6 | 3543.4 | 1.1 |
| *mus81∆*  *yen1∆* | 6 | 2.6 | -1.1 | 6 | 4787.3 | 1.4 |
| *rad1∆* | 6 | 0.79 | -3.5 | 6 | 745 | -4.5 |
| *slx4∆* | 6 | 0.64 | -4.4 | 6 | 1426.7 | -2.3 |

**S3B Table. Median Cas9^D10A^ -induced Ura^-^ recombination frequencies**

|  | **Non-induced** | | | **Induced** | | |
| --- | --- | --- | --- | --- | --- | --- |
| **Genotype** | **n** | **Ura^-^ Frequency (x10^-4^)** | **Fold Change (relative to WT)** | **n** | **Ura^-^ Frequency (x10^-4^)** | **Fold Change**  **(relative to WT)** |
| WT | 34 | 0.29 | N/A | 36 | 1094.5 | N/A |
| *mph1∆* | 6 | 1.4 | 4.8 | 6 | 2038.9 | 1.9 |
| *mus81∆*  *yen1∆* | 6 | 0.33 | 1.1 | 6 | 252.6 | -4.3 |
| *rad1∆* | 6 | 0.33 | 1.1 | 6 | 63.6 | -17.2 |
| *slx4∆* | 6 | 0.52 | 1.8 | 6 | 57.3 | -19.1 |
