# Supplementary material for: The Slx4-Rad1-Rad10 nuclease differentially regulates deletions and duplications induced by a replication fork barrier
Source: PLoS Genet. 2025 May 30;21(5):e1011720. doi: 10.1371/journal.pgen.1011720 (PMC12151478; doi:10.1371/journal.pgen.1011720)
Supplement: S4 Table — All strains are in the W303 background (leu2–3,112 ura2–1 ade2–1 his3–11,15 can1–100). (DOCX) [file pgen.1011720.s011.docx]

**S4 Table. Yeast strains used in this study**

| **Strain** | **Genotype^1^** | **Use** |
| --- | --- | --- |
| LSY2000-2 | *MAT*a *trp1ars1::KanMx6* | Strain construction |
| LSY5156-1 | *MAT*a *trp1ars1::KanMx6 lsb3::5'*Δ*-trp1-KlURA3-trp1-3'*Δ | Strain construction |
| LSY5174-23B | *MAT*a *lsb3::5'*Δ*-trp1-KlURA3-trp1-3'*Δ | Strain construction |
| LSY5231-1 | *MAT*a *lsb3::5'*Δ*-trp1-KlURA3-trp1-3'*Δ *leu2::Gal-TUS-LEU2MX gRNA6-21xTer* | Strain construction |
| LSY5248-1 | *MAT*a *trp1::HphMX lsb3::5'*Δ*-trp1-KlURA3-trp1-3'*Δ | Recombination assay, strain construction |
| LSY5247-1 | *MAT*a *trp1::HphMX lsb3::5'*Δ*-trp1-KlURA3-trp1-3'*Δ *leu2::Gal-TUS-LEU2MX gRNA6-21xTer* | Recombination assay |
| LSY5303-6B | *MAT*α *trp1::HphMX lsb3::5'*Δ*-trp1-KlURA3-trp1-3'*Δ *leu2::Gal-TUS-LEU2MX gRNA6-21xTer* | Recombination assay |
| LSY5303-4D | *MAT*a *trp1::HphMX lsb3::5'*Δ*-trp1-KlURA3-trp1-3'*Δ *leu2::Gal-TUS-LEU2MX gRNA6-21xTer* | Recombination assay |
| LSY5291-18D | *MAT*a *trp1::HphMX lsb3::5'*Δ*-trp1-KlURA3-trp1-3'*Δ *leu2::Gal-TUS-LEU2MX gRNA6-21xTer rad52::LEU2* | Recombination assay |
| LSY5276-10D, 22B | *MAT*a *trp1::HphMX lsb3::5'*Δ*-trp1-KlURA3-trp1-3'*Δ *leu2::Gal-TUS-LEU2MX gRNA6-21xTer rad51::HIS3* | Recombination assay |
| LSY5276-8B | *MAT*α *trp1::HphMX lsb3::5'*Δ*-trp1-KlURA3-trp1-3'*Δ *leu2::Gal-TUS-LEU2MX gRNA6-21xTer rad51::HIS3* | Recombination assay |
| LSY5541-4 | *MAT*a *trp1::HphMX lsb3::5'*Δ*-trp1-KlURA3-trp1-3'*Δ *leu2::Gal-TUS-LEU2MX gRNA6-21xTer rad59::KanMX* | Recombination assay |
| LSY5279-19D | *MAT*a *trp1::HphMX lsb3::5'*Δ*-trp1-KlURA3-trp1-3'*Δ *leu2::Gal-TUS-LEU2MX gRNA6-21xTer rad51::HIS3 rad59::LEU2MX* | Recombination assay |
| LSY5344-19D, 24D | *MAT*α *trp1::HphMX lsb3::5'*Δ*-trp1-KlURA3-trp1-3'*Δ *leu2::Gal-TUS-LEU2MX gRNA6-21xTer rad5::KanMX* | Recombination assay |
| LSY5366-9A, 21C | *MAT*α *trp1::HphMX lsb3::5'*Δ*-trp1-KlURA3-trp1-3'*Δ *leu2::Gal-TUS-LEU2MX gRNA6-21xTer mph1::KanMX* | Recombination assay |
| LSY5361-10A | *MAT*α *trp1::HphMX lsb3::5'*Δ*-trp1-KlURA3-trp1-3'*Δ *leu2::Gal-TUS-LEU2MX gRNA6-21xTer rad5::KanMX mph1::KanMX* | Recombination assay |
| LSY5277-8D, 20D | *MAT*a *trp1::HphMX lsb3::5'*Δ*-trp1-KlURA3-trp1-3'*Δ *leu2::Gal-TUS-LEU2MX gRNA6-21xTer mre11::HIS3MX* | Recombination assay |
| LSY5346-24A | *MAT*α *trp1::HphMX lsb3::5'*Δ*-trp1-KlURA3-trp1-3'*Δ *leu2::Gal-TUS-LEU2MX gRNA6-21xTer mre11::LEU2* | Recombination assay |
| LSY5345-7B | *MAT*a *trp1::HphMX lsb3::5'*Δ*-trp1-KlURA3-trp1-3'*Δ *leu2::Gal-TUS-LEU2MX gRNA6-21xTer* *xrs2::KanMX* | Recombination assay |
| LSY5278-16B, 18A | *MAT*a *trp1::HphMX lsb3::5'*Δ*-trp1-KlURA3-trp1-3'*Δ *leu2::Gal-TUS-LEU2MX gRNA6-21xTer sae2::KanMX* | Recombination assay |
| LSY6123-3C | *MAT*a *trp1::HphMX lsb3::5'*Δ*-trp1-KlURA3-trp1-3'*Δ *leu2::Gal-TUS-LEU2MX gRNA6-21xTer mre11-H125N* | Recombination assay |
| LSY5595-2 | *MAT*a *trp1::HphMX lsb3::5'*Δ*-trp1-KlURA3-trp1-3'*Δ *leu2::Gal-TUS-LEU2MX gRNA6-21xTer dnl4::KanMX* | Recombination assay |
| LSY5596-1 | *MAT*a *trp1::HphMX lsb3::5'*Δ*-trp1-KlURA3-trp1-3'*Δ *leu2::Gal-TUS-LEU2MX gRNA6-21xTer tel1::KanMX* | Recombination assay |
| LSY5303-21C | *MAT*a *trp1::HphMX lsb3::5'*Δ*-trp1-KlURA3-trp1-3'*Δ *leu2::Gal-TUS-LEU2MX gRNA6-21xTer* exo*1::KanMX* | Recombination assay |
| LSY5322-19A | *MAT*α *trp1::HphMX lsb3::5'*Δ*-trp1-KlURA3-trp1-3'*Δ *leu2::Gal-TUS-LEU2MX gRNA6-21xTer exo1::KanMX* | Recombination assay |
| LSY5322-12C | *MAT*α *trp1::HphMX lsb3::5'*Δ*-trp1-KlURA3-trp1-3'*Δ *leu2::Gal-TUS-LEU2MX gRNA6-21xTer sgs1::HIS3* | Recombination assay |
| LSY5303-3C | *MAT*a *trp1::HphMX lsb3::5'*Δ*-trp1-KlURA3-trp1-3'*Δ *leu2::Gal-TUS-LEU2MX gRNA6-21xTer exo1::KanMX* *sgs1::HIS3* | Recombination assay |
| LSY5644-6C, 8B | *MAT*α *trp1::HphMX lsb3::5'*Δ*-trp1-KlURA3-trp1-3'*Δ *leu2::Gal-TUS-LEU2MX gRNA6-21xTer slx4::HIS3* | Recombination assay |
| LSY5372-9C, 21A | *MAT*α *trp1::HphMX lsb3::5'*Δ*-trp1-KlURA3-trp1-3'*Δ *leu2::Gal-TUS-LEU2MX gRNA6-21xTer mus81::KanMX* | Recombination assay |
| LSY5304-17C | *MAT*a *trp1::HphMX lsb3::5'*Δ*-trp1-KlURA3-trp1-3'*Δ *leu2::Gal-TUS-LEU2MX gRNA6-21xTer yen1::HIS3* | Recombination assay |
| LSY5304-15C | *MAT*a *trp1::HphMX lsb3::5'*Δ*-trp1-KlURA3-trp1-3'*Δ *leu2::Gal-TUS-LEU2MX gRNA6-21xTer mus81::KanMX yen1::HIS3* | Recombination assay |
| LSY5305-5D | *MAT*a *trp1::HphMX lsb3::5'*Δ*-trp1-KlURA3-trp1-3'*Δ *leu2::Gal-TUS-LEU2MX gRNA6-21xTer mus81::KanMX yen1::HIS3* | Recombination assay |
| LSY5594-1 | *MAT*a *trp1::HphMX lsb3::5'*Δ*-trp1-KlURA3-trp1-3'*Δ *leu2::Gal-TUS-LEU2MX gRNA6-21xTer slx1::KanMX* | Recombination assay |
| LSY5393-12C | *MAT*a *trp1::HphMX lsb3::5'*Δ*-trp1-KlURA3-trp1-3'*Δ *leu2::Gal-TUS-LEU2MX gRNA6-21xTer rad1::HphMX* | Recombination assay |
| LSY5741-19A | *MAT*a *trp1::HphMX lsb3::5'*Δ*-trp1-KlURA3-trp1-3'*Δ *leu2::Gal-TUS-LEU2MX gRNA6-21xTer scc1-73* | Recombination assay |
| LSY5797-1 | *MAT*a *trp1::HphMX lsb3::5'*Δ*-trp1-KlURA3-trp1-3'*Δ *leu2::Gal-TUS-LEU2MX gRNA6-21xTer tof1::KanMX* | Recombination assay |
| LSY5798-1 | *MAT*a *trp1::HphMX lsb3::5'*Δ*-trp1-KlURA3-trp1-3'*Δ *leu2::Gal-TUS-LEU2MX gRNA6-21xTer csm3::KanMX* | Recombination assay |
| LSY5598-11B | *MAT*α *trp1::HphMX lsb3::5'*Δ*-trp1-KlURA3-trp1-3'*Δ *leu2::Gal-TUS-LEU2MX gRNA6-21xTer rad1::HphMX rad51::HIS3* | Recombination assay |
| LSY5649-7C | *MAT*α *trp1::HphMX lsb3::5'*Δ*-trp1-KlURA3-trp1-3'*Δ *leu2::Gal-TUS-LEU2MX gRNA6-21xTer rad1::HphMX rad52::LEU2* | Recombination assay |
| LS6007-32A | *MAT*α *trp1::HphMX lsb3::5'*Δ*-trp1-KlURA3-trp1-3'*Δ *leu2::Gal-TUS-LEU2MX gRNA6-21xTer mre11::HIS3MX rad51::HIS3* | Recombination assay |
| LS6007-32B, 56B | *MAT*a *trp1::HphMX lsb3::5'*Δ*-trp1-KlURA3-trp1-3'*Δ *leu2::Gal-TUS-LEU2MX gRNA6-21xTer mre11::HIS3MX rad51::HIS3* | Recombination assay |
| LSY6070-1 | *MAT*a *trp1::HphMX lsb3::5'*Δ*-trp1-KlURA3-trp1-3'*Δ *leu2::Gal-TUS-LEU2MX gRNA6-21xTer his3::P_ACT1_-EXO1-T_CYC1_-HIS5MX* | Recombination assay |
| LSY6071-1 | *MAT*α *trp1::HphMX lsb3::5'*Δ*-trp1-KlURA3-trp1-3'*Δ *leu2::Gal-TUS-LEU2MX gRNA6-21xTer mre11::HIS3MX his3::P_ACT1_-EXO1-T_CYC1_-HIS5MX* | Recombination assay |
| LSY5883-8C | *MAT*a *trp1::HphMX lsb3::5'*Δ*-trp1-KlURA3-trp1-3'*Δ *leu2::Gal-TUS-LEU2MX gRNA6-21xTer bar1::trp1* | ChIP-qPCR |
| LSY5897-24A | *MAT*a *trp1::HphMX lsb3::5'*Δ*-trp1-KlURA3-trp1-3'*Δ *leu2::Gal-TUS-LEU2MX gRNA6-21xTer bar1::trp1 mre11::HIS3MX* | ChIP-qPCR |
| LSY5950-1 | *MAT*a *trp1::HphMX lsb3::5'*Δ*-trp1-KlURA3-trp1-3'*Δ *his3::Cas9^D10A^-ER-HIS5MX leu2::ARS607gRNA6-LEU2MX* | Recombination assay |
| LSY5976-19B | *MAT*α *trp1::HphMX lsb3::5'*Δ*-trp1-KlURA3-trp1-3'*Δ *his3::Cas9^D10A^-ER-HIS5MX leu2::ARS607gRNA6-LEU2MX* | Recombination assay |
| LSY6002-47D | *MAT*α *trp1::HphMX lsb3::5'*Δ*-trp1-KlURA3-trp1-3'*Δ *his3::Cas9^D10A^-ER-HIS5MX leu2::ARS607gRNA6-LEU2MX* *mph1::KanMX* | Recombination assay |
| LSY5995-12D | *MAT*α *trp1::HphMX lsb3::5'*Δ*-trp1-KlURA3-trp1-3'*Δ *his3::Cas9^D10A^-ER-HIS5MX leu2::ARS607gRNA6-LEU2MX* *rad1::HphMX* | Recombination assay |
| LSY6254-8C | *MATa trp1::HphMX lsb3::5'*Δ*-trp1-KlURA3-trp1-3'*Δ *his3::Cas9^D10A^-ER-HIS5MX leu2::ARS607gRNA6-LEU2MX* *slx4::HIS3* | Recombination assay |
| LSY5980-6C | *MAT*α *trp1::HphMX lsb3::5'*Δ*-trp1-KlURA3-trp1-3'*Δ *his3::Cas9^D10A^-ER-HIS5MX leu2::ARS607gRNA6-LEU2MX* *mus81::KanMX yen1::HIS3* | Recombination assay |
| LSY6222-1 | *MAT*a *trp1::HphMX lsb3::5'*Δ*-trp1-KlURA3-trp1-3'*Δ *leu2::Gal-TUS-LEU2MX gRNA6-21xTer rad10::KanMX* | Recombination assay |
| LSY6223-1 | *MAT*a *trp1::HphMX lsb3::5'*Δ*-trp1-KlURA3-trp1-3'*Δ *leu2::Gal-TUS-LEU2MX gRNA6-21xTer msh3::KanMX* | Recombination assay |
| LSY6238-3B | *MAT*a *trp1::HphMX lsb3::5'*Δ*-trp1-KlURA3-trp1-3'*Δ *leu2::Gal-TUS-LEU2MX gRNA6-21xTer rad1::HphMX slx4::HIS3* | Recombination assay |

^1^ All strains are in the W303 background (*leu2-3,112 ura2-1 ade2-1 his3-11,15 can1-100*).
