# Supplementary material for: The Slx4-Rad1-Rad10 nuclease differentially regulates deletions and duplications induced by a replication fork barrier
Source: PLoS Genet. 2025 May 30;21(5):e1011720. doi: 10.1371/journal.pgen.1011720 (PMC12151478; doi:10.1371/journal.pgen.1011720)
Supplement: S5 Table — (DOCX) [file pgen.1011720.s012.docx]

**S5 Table. Oligonucleotides**

| **Name** | **Sequence (5′-3′)** |
| --- | --- |
| oLea67-short | CGACTCACTATAGGGCGAATTGG |
| MT30 | GGTATGGTGCACTCCTCGACG |
| MT03 | AGTAAATGATGAACAAATTAAGGGAGAACTTTTTTAAAGGGTGCCCAATAGA  AAGAGAAC |
| MT04 | AAATAAAACGAGCTCTCGAGAACCCTTAATCCTGATGCGGTATTTTCTCC |
| MT05 | TGATCTGTTGTATTGGGATCTCTAGACCTAGAGAGTGCACCATAAACGAC |
| MT06 | TAGGTCTAGAGATCCCAATACA |
| MT08 | TTAAGGGTTCTCGAGAGCTC |
| 5’ ChrVI F | TTAATTGTTTATTACTAGTTAGTAAATGATGAACAAATTA |
| 3’ ChrVI R | ACTCGGCCGCATCTGGAAGTTCAACATGAAAGAGTATAAA |
| 5’ ChrVI extension | ATCTTCGCTGCTTTTCTTTATTAATTGTTTATTACTAGTTAGTAAATGATGAAC  AAATTA |
| 3’ ChrVI extension | AGCACCCTGTTTGCTCGCCGACTCGGCCGCATCTGGAAGTTCAACATGAAA  GAGTATAAA |
| MK173 | TTTACTTCCAGATGCGGCCGAGTGTTTTAGAG |
| MK174 | CTAGCTCTAAAACACTCGGCCGCATCTGGAAGTAAA |
| MT42long | GTTCTAGATTTCTTCGATAGTTTATACTCTTTCATGTTGAACTTCCAGATGC  GGCCGCGACTCACTATAGGGCGAATTGG |
| MT41 | GTTCTAGATTTCTTCGATAGTTTATACTCTTTCA |
| MT39 | GCGGCGCAATTGAAATCAATACG |
| Q5 D10A-2 F | TAGTCCCGATagCCAGTCCGATAGAATACTTCTTGTC |
| Q5 D10A-2 R | ATAGCGTCGGGTGGGCCG |
| ChIP-qPCR-F1 | GTTCTAGATTTCTTCGATAGTTT |
| ChIP-qPCR-R1 | AAGATGACATAATAGTTATTGATGC |
