# Supplementary material for: The Slx4-Rad1-Rad10 nuclease differentially regulates deletions and duplications induced by a replication fork barrier
Source: PLoS Genet. 2025 May 30;21(5):e1011720. doi: 10.1371/journal.pgen.1011720 (PMC12151478; doi:10.1371/journal.pgen.1011720)
Supplement: Attachment — Submitted filename: Response to Reviewers.pdf [file pgen.1011720.s016.pdf]

PGENETICS-D25-00176 Response to Reviewers

We thank the reviewers for their positive evaluation of our manuscript and helpful suggestions to improve clarity of the text and to reinforce conclusions of the study.

Reviewer #1: The Symington lab has carried out an excellent study of copy number variation induced by a conditional replication fork block. The mechanism(s) by which deletions and tandem duplications arise appear to have several genetic distinctions, which likely rules out that most of the events are not simply the result of an unequal sister chromatid exchange (but this could be made more clear). There are many factors at play: Mre11, Exo1, cohesin, but the authors emphasize the role of Slx4 and the Rad1-Rad10 nuclease that appears here to prevent the deletion outcomes. The authors also find that a Cas9-directed nick has outcomes distinctly different from the fork block, most notably with Rad1. Overall the data are clearly presented, though I had a question about the statistical method used.

Other concerns are listed here

l. 130. How large is each Ter repeat? And – though I am not asking for more experiments – is there any insight as to how close to the Ter repeats the stalling occurs and what effect the distance to the recombination construct might have?

The *Ter* repeats are 21-bp in length and are separated by spacers of 15-20 bp. We did not try moving the *Ter* repeats further from the *trp1* reporter in this study. We did insert an array of *Ter* sites upstream of the *trp1* reporter but did not find an increase in the frequency of $Trp^+$ recombinants with Tus expression.

It isn't clear to me that the fork will be blocked only after the reporter 3' TRP-URA3-5' TRP construct has been fully copied. If the block happens sooner, so that perhaps only the 3' TRP portion has been copied, how might that affect the ratio of outcomes?

The reviewer raises the interesting point that stalling might occur without copying the entire reporter. Because homology at the 3' end of the second repeat is required for invasion the upstream repeat to generate a $Trp^+$ recombinant, stalling would need to occur within ~ 500 bp upstream of the Ter array. A $Ura^-$ recombinant could be formed if stalling occurred prior to copying the second repeat. Since the frequencies of these two classes of events are similar in wild-type cells, we assume that both repeats are copied most of the time. If fork stalling and reversal occurred ~ 140 bp before the Tus/Ter block, then there would be no heterology at the end of the *3' trp1* repeat to be removed before priming synthesis after invasion of the 5' repeat. This point has now been added to the Discussion.

l. 170. Is there any cell cycle delay caused by the stalled fork? Fig. S3 doesn't show later time points where a delay might be evident.

The first paper by the Hickson lab reporting the use of Tus/*Ter* to perturb replication fork progression showed no cell cycle delay by FACS and no sensitivity to Tus expression in replication checkpoint mutants.

l. 253. How is the scc1 mutant conditional and how was it assayed?

As noted in the Methods, the *scc1-73* mutation is temperature sensitive and assays were performed at the permissive temperature of 25 °C.

Figures: I am unclear what a Bonferroni "post test" is, or how p<0.05 would still be significant in the case of multiple comparisons. I thought the criteria for significance should drop to a lower p value.

A one-way ANOVA test can be used to determine whether there is a statistically significant difference between the means of two or more independent groups. This test was used to compare mean values of groups of strains under uninduced or induced conditions. The Bonferroni post-test was used to show which specific pairs of means are significantly different, for example, between wild type and mutant x after Tus induction. The p values shown are from the Bonferroni post-test. We also analyzed the data by a Mann-Whitney U test comparing median values and most of the p values obtained were the same as using the ANOVA/Bonferroni post-test. The ANOVA/Bonferroni test of the data was generally more stringent than the Mann Whitney test. For example, the *mus81* single mutant was significantly different to WT (p value of <0.05) by the Mann Whitney test but not by ANOVA/Bonferroni. For the mutants emphasized in this study, the p values were <0.005 for all comparisons with WT by both statistical tests.

Fig. 6. A. I thought that a structure-specific endonuclease would convert the reversed fork to a one-ended structure but with a light blue double-stranded end (yielding a structure similar to that shown in S5). I found this figure confusing. Maybe one needs to distinguish between HJ-cleaving SSEs and one that cleave a single strand (Rad1-10 and others).

Thanks for pointing out this error in the Figure 6A, it has now been redrawn.

Fig. S5. Could this replication require BIR factors?

We tested *pol32* and *pif1* but did not observe a significant decrease in the frequency of Tus-induced recombinants.

Fig. 7. It took quite a while for me to understand Fig. 7, which is the crux of the paper. First of all, the step shown below the labels (e.g. "removal of 3' heterologous end") are actually the substrates for that specific step, rather than the outcome. Towards the end of the Figure there should be an "and" inserted to make it clear these are the two outcomes of a single replication event.

We have modified Fig 7 and the legend to clarify the steps involved.

Although it will make the figure a bit more dense, I think the TRP-URA3 repeats have to be shown instead of the faint gray loop. On the right side of the Figure, the invasion is particularly confusing: the gray line has (I think) zero length and the invasion actually creates a dsDNA loop adjacent to a D-loop. How this gets resolved is not clear to me.

Also, the deletion outcome on the right side is missing.

We added the *trp1 URA3* repeats to the loop out on the left side of Fig 7 and added the deletion outcome on the bottom right. The author is correct that the invasion to form the deletion product would involve loop out of duplex DNA next to the D-loop. We propose that the strands are separated by the replicative helicase from the incoming fork. We recognize that the model is complex but is the only one we could come up with to explain the hyper-deletion and hypo-duplication phenotype of the *rad1, rad10, msh3* and *slx4* mutants.

Moreover, whereas the bottom of the right side shows Rad1-10 is needed to cut the heteroduplex loop at its base (and hence when this doesn't happen, deletions would go up - presumably as a sectored colony), BUT, Rad1-10 is also invoked above, to create the loop in the first place. So, if Rad1-10 is needed here, how can that explain the increase in deletions? This seems to be a problem, but I may have missed the way to resolve it.

We agree that these two functions seem contradictory. We assume that the stringency for Rad1-10 in non-homologous (NH) tail removal is less than in repairing the large loop heterology, perhaps because fork reversal occurs prior to copying the entire reporter so that no NH tail is present (see above).

General: simple unequal crossing-over could generate a TD and a deletion simultaneously. This possibility doesn't get much attention, though I understand that the different effects of various mutants rule out that the majority of events occur in this way. Long-tract GC is also introduced, but again, whether some of these events arise in that way isn't dealt with except to indicate it could happen and might occur more without sister-chromatid cohesion. LTGC is a special form of BIR. As noted above, it might be good to see how pif1 or pol32 affect these outcomes.

The reviewer is correct in suggesting an unequal crossover between sister chromatids could generate both classes of recombinants. Indeed, the suppression of both classes of recombinants by Sgs1 and requirement for HJ resolvases is consistent with a crossover model. However, the requirement for Mph1 for 80% of events suggests that fork remodeling is needed to provide an end for strand invasion. Since the *mph1* mutant generally exhibits a hyper-crossover phenotype in other recombination assays, one would expect an increase in recombinants in the *mph1* mutant if they formed by unequal exchange. The effects of *msh3, rad1* and *slx4* mutations are also more difficult to explain

by an unequal exchange mechanism. We have added an additional paragraph to the Discussion to explain the reasoning behind the models proposed.

Reviewer #2: Triplett et al have developed a clever in vivo system for characterizing repeat instability in response to replication stress. The replication stress is induced by induced Tus protein binding to an array of Ter sites downstream of the direct repeats. The system is able to detect both duplications (Trp+) and deletions (Ura-) that occur without and with Tus induction, via galactose induction of a pGAL-HA-Tus cassette. The authors generated mutations in several genetic pathways involved in processing replication/recombination intermediates, to identify factors that enhance or suppress duplications and/or deletions following the Tus-mediated replication stress. They found that the Tus/Ter-induced recombination is RAD51- and RAD52-dependent, indicating the generation of strand invasion recombination intermediates. Loss of MRE11 or XRS2 increased recombination, in a RAD52-dependent manner, likely independent of its role in resection (MRE11 nuclease activity not required), in NHEJ or checkpoint activation. scc1-73 and loss of TOF1, both of which compromise sister chromatid cohesion, also increased recombination. Loss of EXO1 reduced recombination, likely due to loss of long range resection, while loss of SGS1 increased recombination. The authors suggest that this is due to the loss of Sgs1 recombination dissolution activity. Loss of MPH1 also reduced recombination, potentially affecting fork reversal and/or D-loop dissociation. Finally, loss of either SLX4 or RAD1 reduced duplication recombinants but increased deletions. These were the only factors that had opposite effects on duplications and deletions. Loss of RAD1 and RAD51 or RAD52 resulted in synergistic decreases in duplication recombinants and the RAD1-dependent hyper-deletion phenotype was RAD51- and RAD52-dependent. The authors tested the effect of fork breakage at the direct repeats on the genetic requirements for duplications and deletions and found that MPH1, MUS81 and YEN1 are dispensable for tandem duplications, in contrast to what was observed with the Tus/Ter system. In this modified system, RAD1 was required for deletion events, again in contrast to the TUS/Ter system. These results provide insights into the formation and processing of different recombination intermediates during replication stress (fork stalls). The authors propose a model for copy number variation (duplication or deletion) at stalled replication forks, highlighting the potential role of RAD1 in promoting duplications and preventing deletions. They also propose models for tandem duplication a broken replication forks (Cas9) and through single strand annealing.

1. The data are convincing and the experiments are carefully done and controlled. My major question is about the role of MSH3 or SAW1, both of which function with Rad1-Rad10 in processing 3’ non-homologous tails in recombination intermediates, through recruitment to 3’ ssDNA flaps. The repeat lengths used here are within the range that would show a Msh2-Msh3 effect in SSA. MSH3 has also been shown to be involved in the large loop repair attributed to Rad1-Rad10 in the models here. Msh2-Msh3 also recruits Rad1-Rad10 to B-/Z-DNA junctions. Understanding the contribution of MSH3 and SAW1 would help support and refine the proposed models.

We tested the *msh3* mutant in the assay and did observe a decrease in the frequency of Tus-induced Trp+ recombinants and an increase in the frequency of Ura- recombinants, similarly to the *rad1* mutant. This finding is consistent with the studies from the Petes lab showing that Msh2-Msh3, like Rad1-Rad10, facilitates repair of large loop heteroduplex loops in meiosis. Additionally, we tested a *rad10* mutant to ensure that it behaves the same as *rad1*, it does. These data are now described in the text and included in the revised Figure 4, and S1 and S2 Tables.

2. Further, Msh2-Msh3 interacts with Sgs1 to recruit it to recombination intermediates with homeologous sequences. MSH2 has also been shown to modulate sister chromatid exchange in the presence of inverted repeats. Increased unequal sister chromosome exchange in SGS1 mutations was partially MSH2-depenednt. Again, the role, if any, for MSH proteins can help refine mechanism and models.

See above.

3. Related – the model appears to be that Slx4 recruits Rad1-Rad10 to the recombination intermediates for processing. Are the rad1Δ and slx4Δ epistatic or synergistic for the effect on duplications or deletions? Are the nCas9-induced deletions also dependent on Slx4, as well as Rad1-Rad10.

We generated a *rad1 slx4* double mutant and found the same frequency of TDs and deletions as the respective single mutants indicating epistasis (new S4 Fig). We also tested nCas9-induced recombination in the *slx4* mutant and found that it behaves similarly to *rad1* (revised Figure 6).

Reviewer #3: This is an excellent paper dedicated to the mechanisms responsible for the copy number variations (CNV) that are of pivotal importance for genomic instability in health and disease. The authors attempted to unravel how replication stress leads to CNV. To this end, they developed an elegant reporter cassette to study genetic controls of CNV upon replication stress in a yeast S. cerevisiae. Specifically, they analyzed recombinational mechanisms leading to duplications or deletions upon replication fork stalling at Tus/Ter complex. Their genetic analysis identified the Mph1 translocase, Exo1 exonuclease, as well as Rad52 & Rad51 recombinases as the key proteins driving CNV formation. Remarkably, Slx4 scaffold protein and Rad1-Rad10 nucleases promoted duplications, but counteracted deletion formation. Altogether, these data led them to propose a new and thought-provoking model of how the repair of the stalled replication fork leads to CNV. First, locally stalled replication fork undergoes reversal driven by the Mph1 translocase, which is followed by Exo1 end-resection and strand invasion driven by Rad52 and Rad51. Second, DNA synthesis at the resultant D-loop followed by its subsequent resolution by the convergent replication fork leads to the formation of a specific heteroduplex intermediate flanked by the large loop. Third, resolution of this loop by the Rad1-Rad10 complex promotes duplications while eliminates deletions.

The experimental design is excellent, the results are clear-cut and their interpretations are sound. Nothing to add or subtract. I was particularly impressed by their model involving large-loop heteroduplex intermediates, which made me to rethink some of my own data.

We thank the reviewer for the positive evaluation of our manuscript and interest in the models proposed.
